# Evaluating the Link between BAFF System Gene Expression and Acute Rejection Development in Kidney Transplantation

**DOI:** 10.3390/jcm11143956

**Published:** 2022-07-07

**Authors:** Rafael Alfaro, Santiago Lorente, Víctor Jimenez-Coll, Helios Martínez-Banaclocha, José Antonio Galián, Carmen Botella, María Rosa Moya-Quiles, Manuel Muro-Pérez, Jesús de la Peña-Moral, Alfredo Minguela, Isabel Legaz, Manuel Muro

**Affiliations:** 1 Immunology Services, University Clinical Hospital, Virgen de la Arrixaca-Biomedical Research Institute of Murcia (IMIB), 30100 Murcia, Spain; raf.hellin@gmail.com (R.A.); victorjcoll@gmail.com (V.J.-C.); heliosmar@live.com (H.M.-B.); diegoarmandogalian@hotmail.com (J.A.G.); carmen.botellam@gmail.com (C.B.); rosa.moya2@carm.es (M.R.M.-Q.); muro_manuel@live.com (M.M.-P.); alfredo.minguela@carm.es (A.M.); 2 Nephrology Services, University Clinical Hospital, Virgen de la Arrixaca-Biomedical Research Institute of Murcia (IMIB), 30100 Murcia, Spain; sllorentev@telefonica.net; 3 Pathology Services, University Clinical Hospital, Virgen de la Arrixaca-Biomedical Research Institute of Murcia (IMIB), 30100 Murcia, Spain; jesusdelap@gmail.com; 4 Department of Legal and Forensic Medicine, Faculty of Medicine, Biomedical Research Institute (IMIB), Regional Campus of International Excellence “Campus Mare Nostrum”, University of Murcia, 30100 Murcia, Spain

**Keywords:** BAFF system, gene expression, acute rejection, kidney transplant, forensic pathology

## Abstract

B-cell activating factor (BAFF) system signaling is critical for B-cell homeostasis, effector functions, and tolerance maintenance in transplants, but it has not been studied in kidney transplant recipients (KTRs). The aim was to analyze the changes in BAFF system expression in KTRs with/without acute rejection (AR/NAR). The BAFF system expression was analyzed by qPCR in 40 KTRs. A meta-analysis of BAFF system expression and histological renal damage was identified by the Chronic Allograft Damage Index (CADI) and performed from the GEO database. Proliferation-inducing ligand (APRIL) expression increased at three- and six-months post-KT (*p* = 0.014 and *p* < 0.001). B-cell maturation antigen (BCMA) expression increased at six-months post-KT (*p* = 0.038). BAFF expression remained stable in NAR-KTRs, but was increased in CADI concerning the No-CADI group at one year (*p* = 0.008). BCMA expression increased in the CADI group at one- (*p* = 0.001) and six-years post-KT (*p* = 0.024). At three months, the transmembrane activator and calcium modulator interactor (TACI) gene significantly elevated KTRs with DSAs (donor-specific antibody; *p* = 0.034). KTRs with DSAs significantly increase the B-cell activating factor receptor (R-BAFF; *p* = 0.021) and TACI (*p* = 0.018) between pre- and three-month post-KT. Changes in the expression of the BAFF system increase during post-KTR in the development of AR and chronic allograft damage, and could be an important pathological tool to detect and prevent kidney graft outcomes.

## 1. Introduction

B-cells play an essential role in the biological processes that mediate kidney graft rejection and tolerance through various effector mechanisms, mainly the production of antibodies, the presentation of antigens to T cells, and the secretion of cytokines [1]. The availability of cytokines that offer survival signals, such as IL-7 or BAFF, throughout the formation and proliferation of B-cells is critical for the correct immunological response mediated by B-cells [2]. BAFF signaling plays an essential role in the homeostasis of B-cells and their effector functions, which is fundamental in maintaining tolerance.

BAFF and APRIL are TNF family cytokines with a homotrimeric type II transmembrane structure [1]. The BAFF and APRIL membrane versions are proteolytically digested in consensus sequences by furins, a type of protease, to produce their soluble forms. R-BAFF, TACI, and BCMA are the three receptors in the BAFF system. BAFF can also bind to R-BAFF, also known as TNFRSF13C [3]. B-cells are the primary source of R-BAFF and R-APRIL. R-BAFF and R-APRIL can detach from the cellular membrane, act as soluble forms, and act as a negative feedback mechanism, suppressing B-cell-mediated immune responses by blocking the critical BAFF and APRIL actions [4,5]. The soluble forms of the BAFF receptors are still poorly studied. In multiple myeloma, the serum elevation of sBCMA correlates with the number of plasma cells and clinical status, whereas in lupus erythematosus, sBCMA and sTACI also correlate with the disease activity [6,7]. There are still no studies regarding soluble receptors in kidney transplants (KT), but they could be potential biomarkers of humoral responses to grafts [8].

The patients with higher post-transplantation levels of BAFF have been related to antibody-mediated rejection (AMR). This fact may produce a dysregulation in the microenvironment that promotes the expansion and activation of alloreactive B-cells [9,10]. Therefore, the different molecule proteins of the BAFF system can be helpful as a post-transplant prognostic factor or biomarker, and may notify improvements in immunosuppression [9,11].

Numerous single nucleotide polymorphisms (SNPs) of the BAFF system genes related to B-cells activation and survival have also been investigated [12]. Other points that could also have important repercussions would be the BAFF levels that have been associated with de novo donor-specific antibodies (DSA) development and AMR [13]. Transcriptomics studies in tolerant receptors have also shown increased gene expression related to B-cells [14,15]. Altogether, these analyses can help discover expression profiles that allow recipients to be stratified according to the immunological risk, and differentiate the type of rejection, providing valuable information to the clinician [16].

The aim was to analyze the changes in BAFF system expression in pre- and post-KT sin recipients with and without rejection (AR/NAR, respectively), and study, in silico, their functions in kidney graft outcomes that could be a critical pathological tool to detect and prevent the outcome of kidney grafts.

## 2. Materials and Methods

### 2.1. Demographic Data, Clinical Characteristics, and Study Design

At the University Clinic Hospital “Virgen de la Arrixaca”, 269 adult KT recipients were retrospectively studied (Spain). Luminex DSA determinations, DNA analysis, and complete clinical data were only included in this investigation for recipients whose kidney grafts had been in operation for at least one month after transplantation (patients met all requirements). Return to dialysis was estimated as allograft loss.

Creatinine (0.7 to 1.2 mg/dL) and estimated glomerular filtration rate (eGFR) (>90 mL/min/1.73 m^2^) were measured in all recipients (normal values between brackets). Before the transplant, our patients’ cohorts had the following mean values: creatinine (2.92.1 mg/dL; mean SD).

Finally, a total of 40 patients were selected with equal treatment, exact time of the AR presence, and equality of age and gender for a longitudinal study of monitoring of BAFF system gene expression profiles in KT recipients, consisting of 35 NAR (87.5%) and five AR (12.5%). Of recipients who developed AR, three were antibody-mediated rejection (AMR), and two were acute cellular rejections (ACR). Three recipients (7.5%) received their grafts from living donors, whereas 37 (92.5%) were cadaveric donors. Glomerulonephritis (34.2%), polycystic kidney disease (20.3%), type I diabetes mellitus (11.9%), chronic obstructive pyelonephritis (8.4%), unexplained renal insufficiency (6.1%), lupus nephritis (3.6%), reflux nephropathy (2.4%), and others were the most common reasons for transplantation (13.1%). Regarding induction therapy, 14 (35%) patients received thymoglobulin, whereas five (12.5%) received basiliximab. There were no significant differences in any of the characteristics studied. There were no significant differences in age, sex, HLA incompatibility, or donor type between these patients (Table 1).

Before taking part in the trial, all patients gave informed consent. The study followed the Declaration of Helsinki, and the Ethics Committee approved the HCUVA protocol (PI15/01370 and PI19/01194).

### 2.2. Immunosuppressive Treatment

Oral tacrolimus (Prograf, Astellas, Ireland), mycophenolatemofetil (MMF; CellCept, Roche, Switzerland), and prednisolone were given to all of the participants (Dacortin, Merck, Spain). The tacrolimus (FK)-based protocol was started at a dose of (0.10–0.15 mg/kg/day) and gradually increased to maintain a trough level of FK in whole blood between 8 and 12 ng/mL during the first month after transplant, between 7 and 10 ng/mL during the second and third months, and between 5 and 8 ng/mL after that. MMF was begun at 2000 mg/day and gradually reduced to 1000–1500 mg/day over the first month after surgery, depending on the number of white blood cells.

On the day of transplantation, day 1–2, and day 3–4 after surgery, methylprednisolone was given intravenously in doses of 500, 250, and 125 mg/day, respectively. Oral prednisolone was started at a dose of 20 mg on day five following the transplant and gradually decreased to 5–10 mg/day within 2–3 months.

### 2.3. Kidney Rejection Diagnosis

Allograft ACR was defined as a 20% increase in serum creatinine above baseline and biopsy-proven rejection (specimens were evaluated by light microscopy and immunofluorescence staining with a marker of classical complement activation (C4d) and classified according to the Banff classification, which was updated in 2017) [17]. As previously published, the diagnosis of acute AMR requires distinguishable histopathological findings, positive C4d staining in peritubular capillaries, and the simultaneous presence of DSA [18,19].

Pulsed steroids (500 mg methylprednisolone boluses) and enhanced maintenance immunosuppression were used to treat mild acute cellular rejection (Banff grade I). Anti-thymocyte globulin was used to treat all other ACR (ATG).

Steroid-sensitive rejections (ACR Banff grade I) and steroid-insensitive rejections (ACR Banff grade II and III, and AMR) were the two types of AR episodes. AMR was also treated with pulse steroids and intravenous immunoglobulin (0.25 gr/kg for the first session and 1 gr/kg for the last session, for a total of 140 g divided into two doses) in conjunction with plasmapheresis (three sessions a day, every five days). Anti-CD20 (Rituximab, Roche Pharmaceuticals) was then given intravenously at a dose of 500 mg. Two patients receiving the anti-proteasome inhibitor, Bortezomic (Velcade^®^, formerly PS-341), also received anti-AMR therapy.

### 2.4. Design of the Study of Gene Expression of Molecules of the BAFF System

**Figure 1:**
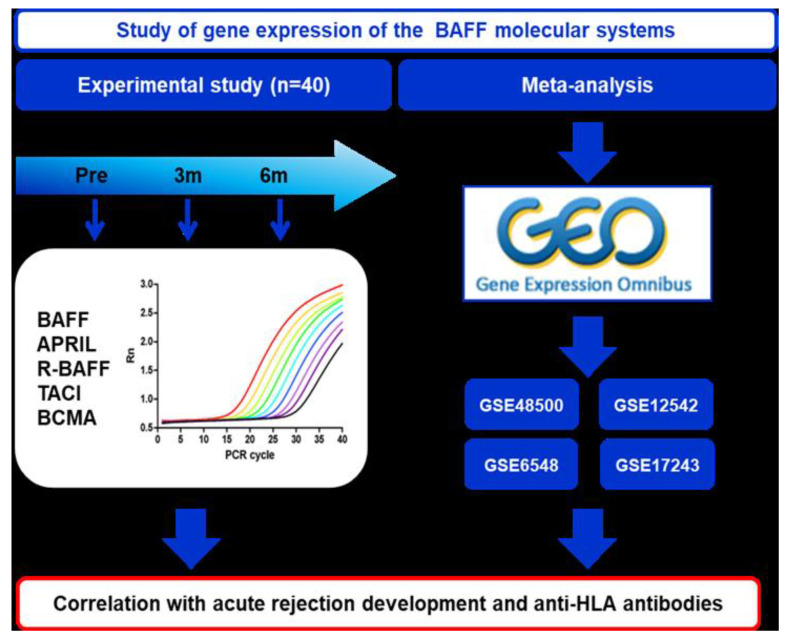
Design of gene expression study of BAFF system molecules in kidney recipients. AFF, B-cell activating factor; APRIL, Proliferation-inducing ligand; R-BAFF, B-cell activating factor receptor; TACI, transmembrane activator and calcium modulator interactor; BCMA, B-cell maturation antigen.

The gene expression study of different members of the BAFF system was divided into two parts, as shown in Figure 1: a longitudinal study and a meta-analysis of transcriptomics studies performed on kidney transplant recipients (KTRs). The longitudinal study was carried out with samples of KTRs obtained pre- and at three- and six-months post-transplantation, where we measured the gene expression of BAFF system molecules (BAFF, APRIL, R-BAFF, TACI, and BCMA) by quantitative PCR. On the other hand, we performed a meta-analysis of transcriptomic studies of KTRs obtained from the public GEO database (Gene Expression Ómnibus) [18,19], focusing on the analysis of gene expression of the BAFF system. The data obtained from the experimental study and the meta-analysis were used to establish the correlation between the levels of transcripts of the BAFF system molecules, AR incidence, and the eventual different DSA production.

### 2.5. Total RNA Extraction

The Maxwell 16 miRNA Kit was used to isolate total RNA from peripheral blood leukocytes according to the manufacturer’s instructions (Promega, Madison, WI, USA). Cell Lysis Solution Genomic Purification was used to lyse peripheral blood samples (Promega, Madison, WI, USA). On a NanoDrop2000, the concentration and purity of RNA were determined (ThermoScientific, Waltham, MA, USA). Pure RNA samples were defined as 260/280 nm ratios between 2.0 and 2.2. As previously reported, RNA integrity was determined using a 1% agarose gel electrophoresis (25). Subsequently, the purified RNA was frozen at −60 °C until the moment of use.

### 2.6. mRNA Reverse Transcription

mRNA was reverse-transcribed to complementary DNA (cDNA) using the RT2FirstStrand Kit (Qiagen, Frederick, MD, USA). Briefly, 1 µg of total RNA was incubated with 2 µL of Buffer GE and RNase-free water to a final volume of 10 µL for 5 min at 42 °C for the removal of genomic DNA. Next, 4 µL of BC3 buffer, 1 µL of Control P2, 2 µL of RE3 Reverse Transcriptase Mix, and RNase-free water were added to the above mixture to a final volume of 20 µL. The mixture was incubated at 42 °C for 15 min. Following incubation, the reverse transcription mixture was diluted in RNase-free water to a final volume of 110 µL. Subsequently, cDNA samples were stored at −20 °C until the moment of use.

### 2.7. Gene Expression of the BAFF System Molecules

The BAFF system’s gene expression was analyzed by quantitative PCR (qPCR). qPCR was carried out using the following TaqMan-type hydrolysis probes (Applied Biosystem, Foster City, CA, USA): *HPRT1*(Hs99999909_m1), *CD19* (Hs00174333_m1), *TNFSF13B* (BAFF; Hs00198106_m1), *TNFSF13* (APRIL, Hs00601664_g1), *TNFRSF13C* (R-BAFF, Hs00606874_g1), *TNFRSF13B* (TACI, Hs00963364_m1), *TNFRSF17* (BCMA, Hs03045080_m1).

The expression of the HPRT1 gene was used as an endogenous control for the normalization of BAFF and APRIL expression, whereas the CD19 gene was used to normalize the expression of R-BAFF, TACI, and BCMA. The relative expression of the transcripts was calculated according to the 2^−ΔΔCt^ method [20]. An ABI-7500 Fast Real-Time PCR System (Applied Biosystem, Singapore) was used for qPCR. A PCR reaction mix was prepared for each qPCR reaction with 10 µL of TaqMan Fast Advanced Master Mix (Applied Biosystem, Foster City, CA, USA), 7 µL of nuclease-free water, and 1 µL of the corresponding TaqMan probe. The reaction mixture was then transferred to a 96-well plate, and 2 µL of cDNA was added for patient samples or 2 µL of nuclease-free water in case of negative controls. Thermal cycler conditions in the real-time PCR were: 1 cycle for incubation for 2 min at 50 °C, and one cycle for initial activation of Taq polymerase during 20 s at 95 °C, and 40 cycles of denaturalization for 3 s at 95 °C and primer union/extension during 30 s at 60 °C.

### 2.8. Meta-Analysis of Transcriptomic Data from the GEO Database

#### 2.8.1. Inclusion Criteria for Gene Expression Studies

A search was carried out in the GEO database (Gene Expression Omnibus), available at https://www.ncbi.nlm.nih.gov/geo/, for studies published until 16 February 2019, using the following keywords: “transplant”, “renal”, “kidney”, “rejection”, “humoral rejection”, “antibody-mediated rejection”, “DSA”, and/or “Homo sapiens”. The inclusion criteria were samples of human origin, samples from biopsies or peripheral blood, and samples from transplant patients with good graft function and a diagnosis of AR. As an exclusion criterion, samples diagnosed with chronic rejection were rejected, as they were not the object of this study.

Five studies met the inclusion criteria (GSE14346, GSE15296, GSE46474, GSE36059, and GSE21374) [21,22,23,24,25]; study GSE50084 was discarded, as it did not provide information that allows differentiating between acute and chronic rejections. The characteristics of the studies included in the meta-analysis are summarized in Supplementary Table S1.

#### 2.8.2. Analysis of GEO Studies

GEO2R [26] is a web tool that compares two data groups from a gene expression study deposited in the GEO database. The samples obtained from each cohort were separated into two groups according to AR incidence. Next, the GEO2R tool was used to obtain the fold change values and the adjusted *p*-value using the FDR (false discovery rate) method of Benjamini and Hochberg for the comparisons made with the expression levels of the TNFSF13B genes (BAFF), TNFSF13 (APRIL), TNFRSF13C (R-BAFF), TNFRSF13B (TACI), and TNFRSF17 (BCMA). The option of logarithmic transformation of the data of the GEO2R tool was kept in “Auto-detect” for all the comparisons.

### 2.9. Statistical Analysis

The mean and standard error of the mean (SEM) were used for quantitative data, whereas for categorical data, percentages were used. Fisher’s exact test, often known as the X2 test, was used to compare categorical variables. The Kolmogorov–Smirnov test was used to ensure that the data were normal. The Mann–Whitney U test was used to compare two groups with variables that were not normalized. The Kruskal–Wallis test and Dunn’s post hoc test with Bonferroni adjustment for multiple comparisons were employed to compare three or more groups. Correlation analyses were carried out using the Spearman index, as previously described [27,28].

The Wilcoxon non-parametric test for related samples was utilized for the longitudinal comparison of two related groups. Three or more similar groups were compared using the Friedman test with Wilcoxon post hoc. The construction of ROC curves was used to assess the sensitivity and specificity of biomarkers. The area under curve was used to assess discriminating capacity (AUC). The Youden index was used to find the appropriate cut-off value that maximizes sensitivity and specificity. In multiple comparisons, the *p*-value was corrected using the Benjamini–Hochberg or Bonferroni methods. For all statistical tests, *p* < 0.05 (or p-corrected 0.05 in the event of multiple comparisons) was considered significant [28]. The graphs and statistical analyses were created using the software packages, Statistical Package for the Social Sciences (SPSS, version 22, Chicago, IL, USA) and GraphPad Prism (version 6, San Diego, CA, USA), as well as the R programming language, which was used in the Integrated Development RStudio version 3.4 environment.

## 3. Results

### 3.1. Dynamics of Gene Expression during the Post-Transplantation Period

**Figure 2:**
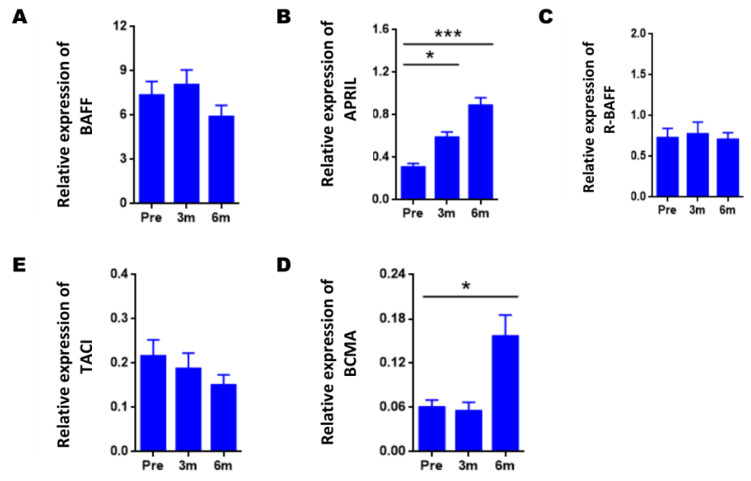
Variation of the gene expression of BAFF, APRIL, and their receptors during post-transplantation. Comparison of the levels of gene expression of BAFF (**A**), APRIL (**B**), R-BAFF (**C**), TACI (**D**), and BCMA (**E**) at three- and six-months post-transplantation vs. pre-transplantation in the total of 40 patients in the pre, 3 m, and 6 m groups. The HPRT gene was used as an endogenous control to normalize BAFF and APRIL gene expression, and the CD19 gene to normalize R-BAFF, TACI, and BCMA gene expression. Expression data were represented as the mean ± SEM. Statistical analyses were performed using the Wilcoxon test for related samples. Values of *p* < 0.05 were considered significant. * *p* < 0.05; *** *p* < 0.001.

Firstly, we study how the expression of genes varies during the post-transplant period compared to the pre-transplant point (Figure 2). BAFF gene expression did not undergo significant changes after transplantation (Figure 2A); however, APRIL expression showed an increase after transplantation, being significant both at three-months (0.31 ± 0.03 vs. 0, 59 ± 0.05; *p* = 0.014) and six-months post-transplantation (0.89 ± 0.06 vs. 0.31 ± 0.03; *p* < 0.001) with respect to pre-transplantation (Figure 2B).

However, no significant differences in R-BAFF and TACI gene expression were found post-transplantation (Figure 2C,E). Finally, the expression of BCMA did show a significant increase at six-months post-transplantation concerning pre-transplantation (0.061 ± 0.009 vs. 0.157 ± 0.028; *p* = 0.038, Figure 2D).

### 3.2. Gene Expression of BAFF, APRIL, and Their Receptors in Kidney Recipients with AR

**Figure 3:**
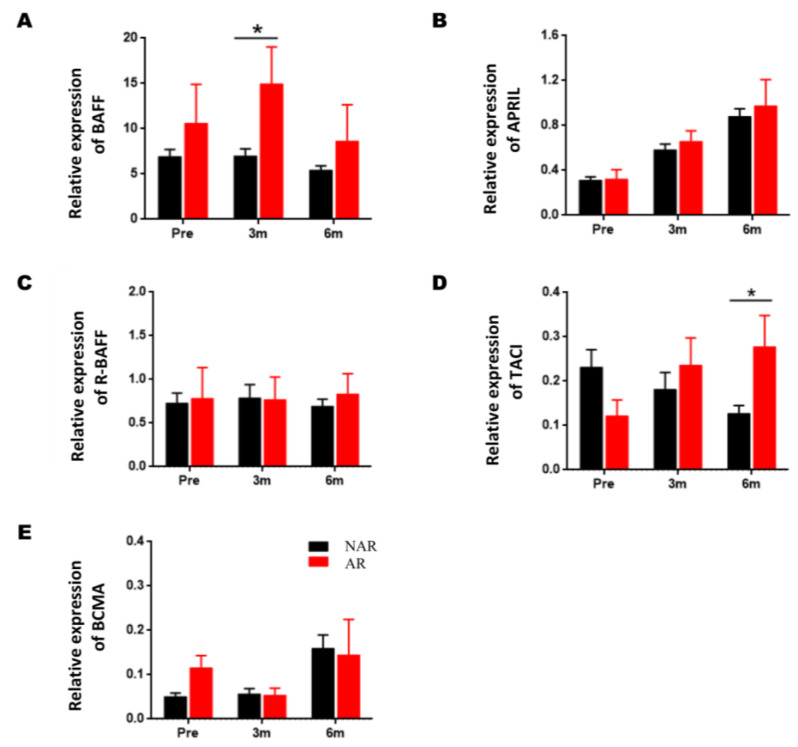
Association of the gene expression of BAFF, APRIL, and their receptors (R-BAFF, TACI, and BCMA) with acute rejection. Comparison of BAFF (**A**), APRIL (**B**), R-BAFF (**C**), TACI (**D**), and BCMA (**E**) gene expression levels in peripheral blood samples among KTRs without (NAR, *n* = 35) and with acute rejection (AR, *n* = 5), obtained pre- and at three- and six-months post-transplantation. The HPRT gene was used as an endogenous control to normalize BAFF and APRIL gene expression, and the CD19 gene to normalize R-BAFF, TACI, and BCMA gene expression. Expression data are represented as the mean ± SEM. Statistical analyses were carried out using the Mann–Whitney U test. Values of *p* < 0.05 were considered significant. * *p* < 0.05.

The gene expression values of the BAFF system molecules and AR incidence are shown in Figure 3. BAFF gene expression remains relatively stable in KTRs from the NAR group, whereas a significant increase is observed three months after KTRs from the AR group (Figure 3A, 14.96 ± 4.06 vs. 6.99 ± 0.78; *p* = 0.047), although at six months post-transplantation, there are no longer significant differences. Its expression increased during the two groups’ post-transplantation period regarding APRIL, with no significant differences (Figure 3B).

Regarding the gene expression of BAFF receptors, R-BAFF remains stable in post-transplantation stages, both in KTRs of both NAR/AR groups, with no significant differences being observed between both groups (Figure 3C). Regarding the TACI expression, we could observe that in the NAR-KTRs, TACI expression decreases compared to pretransplantation, an inverse trend to that observed in the AR-KTRs, where its expression increases at three- and six-months post-transplantation; although, only at six-months post-transplantation were significant differences observed between both groups (Figure 3D, 0.278 ± 0.07 vs. 0.127 ± 0.018; *p* = 0.011). Regarding the expression of BCMA, no significant differences were observed between both groups (Figure 3E).

### 3.3. Association of the Pre-Transplant Expression Levels of the BAFF System with Graft Function

To estimate the association between the pre-transplantation levels of the gene expression of the BAFF system molecules and graft function during post-transplantation, we divided the NAR-KTRs into two groups according to estimated glomerular filtration rate (GFR) obtained at 18-months post-transplant. The KTRs with GFR ≥ 63.2 mL/min/1.73 m^2^ were included in the group with good renal function, whereas the KTRs with GFR ≤ 63.2 mL/min/1.73 m^2^ were included in the group with average/poor kidney function.

**Figure 4:**
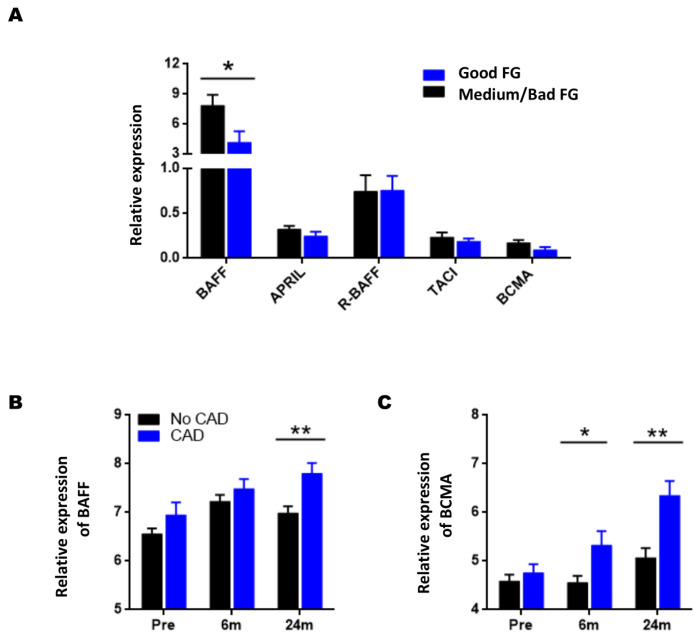
Association of post-transplantation kidney graft function and BAFF system gene expression levels at pre-transplantation (**A**) and BAFF and BCMA Gene Expression in Chronic Graft Damage Study GSE25902. Good GF with 94 and Medium/Bad FG with 26 patients (**B**,**C**). Data are represented as the mean ± SEM. Statistical analyses were performed using the Mann–Whitney U test to compare post-transplant groups. Comparison of the expression levels of BAFF (**B**) and BCMA (**C**) between the groups with chronic graft damage (CAD, blue bars) with 24 patients and those without CAD with 96 patients (black bars) at pre- and 6- and 24-months post-transplantation. Values *p* < 0.05 were considered significant. * *p* < 0.05; ** *p* < 0.01.

Figure 4A shows that KTRs with a good post-transplant evolution have significantly lower BAFF gene pre-transplant expression levels (4.16 ± 1.10 vs. 7.58 ± 1.07, *p* = 0.016). We also observed a decrease in the levels of APRIL (0.245 ± 0.049 vs. 0.321 ± 0.037, *p* = 0.179) and BCMA (0.092 ± 0.034 vs. 0.171 ± 0.033, *p* = 0.085) genes, although the differences were not statistically significant. No significant differences were obtained in the pre-transplantation expression of R-BAFF and TACI genes.

Then, the relationship between the expression of these molecules and chronic histological damage in kidney allografts, defined by the CADI index (Chronic Allograft Damage Index), was also analyzed using the transcriptomic data available from the GSE25902 study obtained from the GEO database. This study’s samples were divided at 24-months post-transplantation into two groups based on their CADI index.

KTRs with a CADI index ≥ 6 were included in the group of chronic graft damage (CAD), and transplants with CADI < 6 as those without chronic damage (No-CAD).

At pre-transplantation, there are no significant differences between patients who progress to CAD and those who do not. When comparing the post-transplant expression levels, we observed that BAFF gene expression levels at 24 months after implantation were significantly higher in the CAD group than in the No-CAD group (Figure 4B, *p* = 0.008).

Regarding the levels of expression of BCMA gene, at six-months post-transplantation, these were significantly higher in the CAD group (*p* = 0.024), with differences that increased at 24-months post-transplantation (*p* = 0.001) (Figure 4C). No significant differences were observed in APRIL, R-BAFF, and TACI gene expression levels.

### 3.4. Gene Expression of BAFF, APRIL, and Their Receptors in GEO Database Studies: Biopsy Samples and Peripheral Blood

Given the small number of samples at the time of rejection, we decided to perform a meta-analysis using gene expression data from transcriptomics studies deposited in the GEO database. To do this, we researched with the criteria established, and finally obtained five studies: three carried out on RNA extracted from peripheral blood (GSE14346, GSE15296, GSE46474), and two on RNA extracted from samples of kidney graft biopsy (GSE36059, GSE21374). From these studies, using the GEO2R web tool, we obtained the gene expression ratios (fold change) and the statistical significance of the BAFF system molecules when comparing samples of KTRs without and with AR.

**Figure 5:**
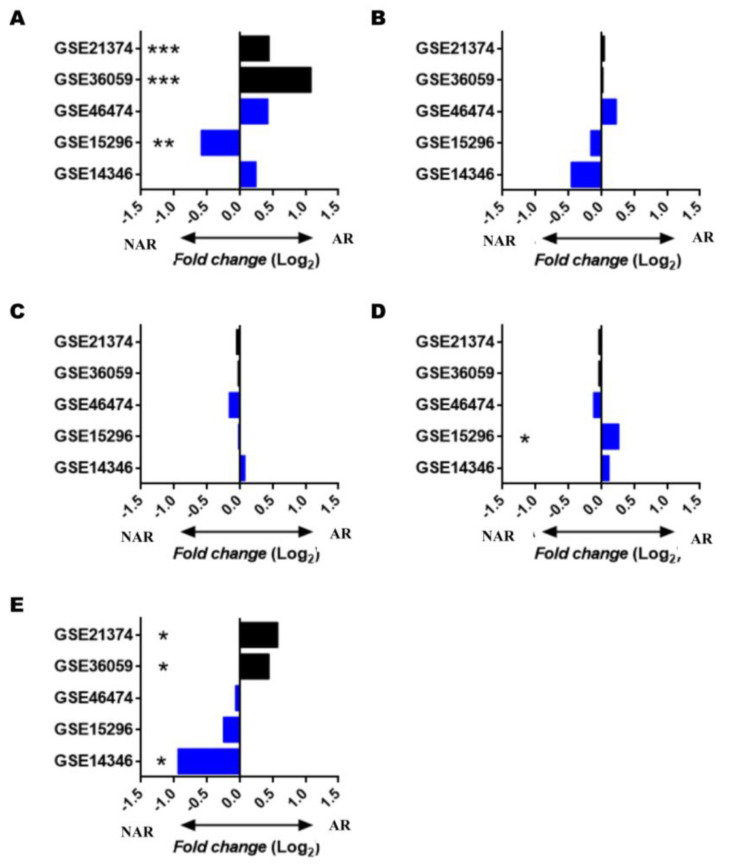
Gene expression of BAFF, APRIL, and their receptors (R-BAFF, TACI, and BCMA) from selected GEO studies. Figures represent the ratio of gene expression (fold change) of BAFF (**A**), APRIL (**B**), R-BAFF (**C**), TACI (**D**), and BCMA (**E**) among KTRs of NAR and AR groups in studies performed in peripheral blood (blue bars) and biopsy samples (black bars). The sample sizes of each analyzed cohort and the number of patients with AR and NAR are shown in Table 1. Positive values indicate increased expression in KTRs from the AR group, and negative values increase in KTRs from the NAR group. AR, acute rejection; NAR, non-acute rejection. *p* values were adjusted by the FDR method. FDR values < 0.05 were considered significant. * FDR < 0.05; ** FDR < 0.01; *** FDR < 0.001.

From the studies derived from biopsies, we could observe that the expression of BAFF was over-expressed in KTRs with AR in the two studies analyzed (Figure 5A, FDR < 0.001). On the other hand, in the case of blood, only in the study GSE15296 were significant differences (FDR = 0.007) observed, and, different to what was observed in biopsy samples, expression levels were increased in patients in the NAR group. Regarding the expression levels of APRIL (Figure 5B), no significant differences were observed in any studies.

Regarding BAFF receptors, it was observed that in both studies derived from biopsies, there was an overexpression of BCMA (Figure 5E) in KTRs of the AR group (FDR = 0.011 and FDR = 0.014). In blood samples, in the study of GSE14346, there was also a significant increase in NAR group (FDR = 0.037). Regarding the TACI receptor (Figure 5D), in GSE15296, a significant increase was observed in the AR group (FDR = 0.012), with no significant differences observed in the rest of studies. No significant differences exist in any studies (Figure 5C) regarding the R-BAFF.

### 3.5. Gene Expression of BAFF, APRIL, and Their Receptors: Influence of the Presence of Anti-HLA and DSA

Of the studies extracted from the GEO database, only the study GSE50084 contains complete information related to DSAs; the other studies were discarded because they did not present enough information on the presence or absence of antibodies in the control samples.

**Figure 6:**
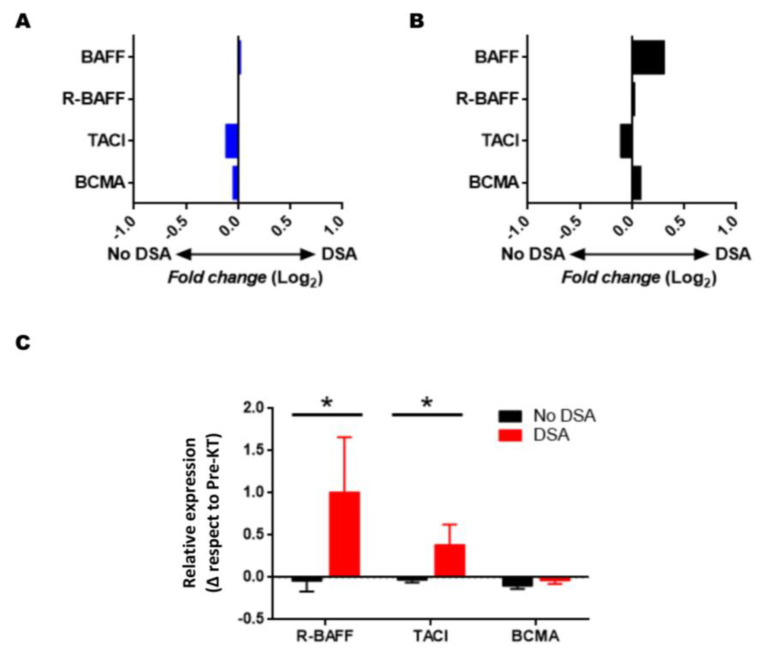
Gene expression of BAFF and its receptors (R-BAFF, TACI, and BCMA) from study GSE50084, and variation of the BAFF gene expression in kidney recipients at three-months post-transplantation. The figure represents the ratio of BAFF, R-BAFF, TACI, and BCMA gene expression in peripheral blood (**A**) and renal biopsy (**B**) in the study GSE50084 among KTRs samples, where positive values indicate an increased expression in transplants with DSA antibodies’ presence, and negative values indicate an increased expression in KTRs with DSA antibodies’ absence. *p* values are adjusted by the FDR method. FDR values < 0.05 were considered significant. (**C**) Variation of the BAFF expression in KTRs at three-months post-transplantation. The figure represents the BAFF system gene expression levels varying between pre- and three-months post-transplantation in KTRs without DSA (No DSA, *n* = 36) and DSA antibodies (*n* = 4) during the follow-up period. Values are represented as the mean ± SEM. Comparisons were made using the Mann–Whitney U test. *p* values < 0.05 were significant. * *p* < 0.05.

Data on APRIL were not included as they were unavailable in this study. The data from this study correspond to samples of KTRs with good renal function, excluding patients with AR to minimize covariates that could influence this analysis. The gene expression data from this study, both in biopsy and peripheral blood (Figure 6), show significant differences in any of the genes studied in our present study.

Later, we analyzed the variation in gene expression between pre-and post-transplantation. In Figure 6C, we observe that KTRs with DSA antibodies have a significant increase in the expression levels of R-BAFF (1.01 ± 0.65 vs. −0.05 ± 0.12; *p* = 0.021) and TACI (0.39 ± 0.24 vs. 0.03 ± 0.02; *p* = 0.018) between pre- and three-months post-transplantation. No significant differences were observed in the rest of BAFF system genes.

**Figure 7:**
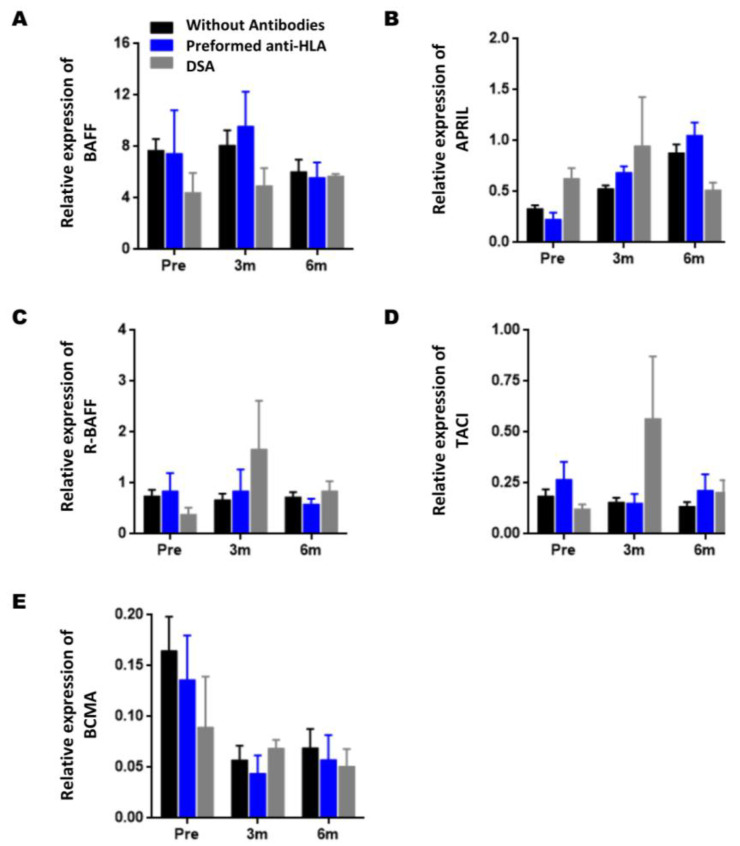
Association of the gene expression of BAFF, APRIL, and their receptors (R-BAFF, TACI, and BCMA) with anti-HLA antibodies status. Comparison of BAFF (**A**), APRIL (**B**), R-BAFF (**C**), TACI (**D**), and BCMA (**E**) gene expression levels in peripheral blood samples among KTRs without anti-HLA antibodies (black, *n* = 30), preformed anti-HLA antibodies (blue, *n* = 6) at pre-transplantation, and with de novo DSA antibodies (gray, *n* = 4). The HPRT gene was used as an endogenous control to normalize BAFF and APRIL gene expression, and the CD19 to normalize R-BAFF, TACI, and BCMA gene expression. Expression data are represented as the mean ± SEM. Statistical analyses were performed using the Kruskal–Wallis test and Dunn’s *post hoc* test with Bonferroni correction for multiple comparisons. Values *p* < 0.05 were considered significant.

In our series, 10 KTRs were positive for anti-HLA antibodies, of which, six were performed without DSA antibodies, four were de novo DSA antibodies, whereas 30 KTRs were negative for anti-HLA antibodies during the follow-up period of this study. The results in Figure 7 show no significant differences between the groups’ expression levels of the studied molecules. Only the TACI gene expression at three-months post-transplantation showed a significant elevation in KTRs with DSA antibodies (*p* = 0.034), but lost statistical significance after correction for multiple comparisons using the Bonferroni method (*p* = 0.101).

## 4. Discussion

This study monitored and analyzed the changes in BAFF system gene expression in KTRs, and studied their kidney graft function in AR and NAR groups. The CADI index also identified a meta-analysis of BAFF system gene expression and histological renal damage performed from the GEO database. Studies of the gene expression of molecules of the BAFF system are scarce in KT.

In this study, we evaluated the levels of BAFF system transcripts in KTRs, and their association with AR development and anti-HLA antibody production. We validated our results in different kidney transplant studies using the GEO database and the GEO2R web application.

First, we studied the dynamics in a post-transplantation period of gene expression of the BAFF system molecules. Our results showed that, after transplantation, the BAFF, R-BAFF, and TACI transcripts remained relatively stable and did not vary concerning pretransplantation. In contrast, APRIL gene expression showed a progressive increase at three- and six-months post-transplantation. BCMA expression showed no variation at three-months post-transplantation, but a sudden rise was observed at six months. Our work is vital because there is no data in the literature on the dynamics of transcripts in kidney transplantation. Xu et al. [29] showed that BAFF mRNA levels in recipients increase from the first year post-transplantation compared to healthy controls and dialysis patients. These data from Xuet al. [29] could suggest that our post-transplant follow-up period, which did not exceed six months in the transcript analysis, was too short to observe any differences.

On the other hand, APRIL is a cytokine mainly expressed by immune system cells, such as macrophages and dendritic cells [30]. It is known that, after transplantation, an immune activation occurs due to cellular damage derived from ischemic times or surgery, among other causes, which can lead to an innate activation that leads to increased APRIL expression [31]. However, this hypothesis should be corroborated in future studies. Our data in the study of the BAFF system’s soluble forms (data not exposed) show that the serum levels of APRIL decrease during the post-transplantation period. Therefore, as a hypothetical element, the increase in the APRIL gene expression could also correspond to a homeostatic mechanism for recovering pre-transplant basal levels.

According to AR incidence, when we divided our cohort into two groups, it was observed that AR recipients have higher levels of BAFF than those of NAR recipients; although, only three months after transplantation were significant differences observed.

Elevated BAFF levels have been associated with AMR. However, it must be taken into account that most studies have been carried out on soluble and non-transcribed BAFF [14,27], so the results may not correspond precisely to the same evaluated reality. The study by Thibault-Espitia et al., shows that high levels of R-BAFF transcripts and low levels of BAFF transcripts have an increased risk of long-term graft dysfunction [9]. Transcriptomics studies have shown the association of high levels of BAFF and BCMA gene expression with biopsy samples with rejection [32]; although, in our study, we did not observe differences in the levels of expression of BCMA in peripheral blood. Although BAFF dysregulation has been associated with antibody-mediated immune responses, this cytokine can act as a costimulator in T cell activation and promote differentiation towards the Th1 phenotype, contributing to inflammatory responses [33,34].

On the other hand, TACI receptor gene expression has opposite tendencies in AR and NAR groups during the post-transplantation period. In patients in the NAR group, a decrease in TACI expression was observed in post-transplant periods, and on the contrary, the AR group observed an increase, obtaining differences at six-months post-transplantation. TACI is expressed mainly in plasmablasts and plasma cells, in which, APRIL/TACI signaling promotes its activation and survival and contributes directly to humoral responses [35]. Animal models show that TACI activation promotes IgG1 secretion and B-cell differentiation to plasmablasts [36].

These discoveries have led to the development of drugs blocking the BAFF system to stop antibody-mediated responses. In this sense, atacicept is a fusion protein that combines the TACI receptor binding domain with a human IgG, with the ability to block both BAFF and APRIL. Different animal models show satisfactory responses when reducing allospecific responses using atacicept [37,38]. Next, we searched the GEO database and evaluated the BAFF system molecules’ gene expression levels in five cohorts of KTRs, with and without AR, from samples from kidney biopsies (GSE21374, GSE36059) and peripheral blood (GSE46474, GSE15296, GSE14346) [21,22,23,24,25].

Analysis using the GEO2R tool shows that BAFF and BCMA levels are over-expressed in the two cohorts with kidney biopsy. Monocytes and macrophages are two of the primary producers of BAFF. Monocyte infiltration is associated with poorer graft survival, linked to BAFF’s increased expression in these cohorts [39].

Furthermore, recent studies show that BAFF expression is not restricted to cells of hematopoietic origin, and renal epithelium can also synthesize it under certain stimuli [40]. During rejection, there is infiltration inside graft of B lineage cells, such as plasmablasts and plasma cells, which would explain the increase in BCMA expression [41,42]. Thaunat et al. [42] showed that the local production of anti-HLA antibodies in tertiary lymphoid organs within the graft maintains allospecific humoral responses during chronic rejection. The increased BCMA in the graft could mean increased B-cell differentiation towards antibody-secreting cells.

In contrast, there are wide discrepancies in the three cohorts with samples obtained from peripheral blood. In both GSE15296 and GSE14346 cohorts, the decrease in BAFF expression was associated with AR, unlike in the biopsy cohorts. The decrease in BAFF and BMCA in peripheral blood observed in these cohorts could be due to massive monocyte and plasmablast migrations from the periphery towards the graft during AR, which would reciprocally increase the expression of these molecules in the allograft. However, we have no data from the same patient, both blood and biopsy, at the time of rejection, so we cannot corroborate our hypothesis.

BAFF over-expression has been associated with an increased risk of production of DSA [9,43,44], although the pediatric cohort of Lehnhardt et al. [22] found no association. In non-human primates, pharmacological blocking of BAFF and APRIL has been shown to prevent DSA antibodies’ formation [37]. However, another study [38] in rodent models indicates that it is inadequate, although both agree on reducing AMR incidence. In our cohort, we did not obtain any association of the expression levels of the BAFF system molecules with anti-HLA antibody production. However, it must be considered that we have observed a low incidence of DSA antibodies, which may have prevented any association from being obtained with the short follow-up period.

For example, Thibault et al. [9] performed a follow-up of more than ten-years post-transplantation, whereas other studies have been carried out on soluble BAFF and not on transcripts [9,43,44]. We used the GSE50084 cohort from the GEO database to confirm our results, and compared the BAFF system molecules’ gene expression levels between biopsy samples with and without DSA [45]. As in our cohort, we did not find any association between them. However, when we wanted to analyze the difference in expression between pre- and post-transplantation, we observed that the KTRs that generate DSAs increase R-BAFF and TACI expression.

These receptors are related to B maturation and are crucial in humoral responses [46]. Increased expression of the receptors can promote B-cells’ survival and maturation, increasing their effector functions and differentiation towards antibody-producing cells. This can promote the expansion of alloreactive B-cells with an increase in DSA antibodies, as we have observed in our series. Although soluble BAFF levels have been proposed as a risk biomarker for DSA antibody development, according to our results, transcript levels are not helpful. However, the evolution of the expression of BAFF receptors could help predict future allospecific humoral responses. This response is critical in all transplantation types, even the most tolerant, such as the liver graft [47].

To study the BAFF system molecules’ relationship with chronic graft damage, we used the GSE25902 cohort [25]. The KTRs were divided on their CADI (Chronic Allograft Damage Index) index two-years post-transplantation. Those with an index CADI > 6 and without AR were classified as having chronic graft damage. The results show that the expression levels of BAFF and BCMA are elevated two years after transplantation in the KTRs with chronic graft damage. In the case of the expression of BCMA, an elevation was observed as early as six-months post-transplantation, so molecular changes were already observable in biopsies months before the damage occurred.

The scarcity of patients with AR and DSA antibodies in our cohort is a limitation of our study. Furthermore, the in silico analyses show a clear association between the BAFF system molecules’ expression levels with chronic graft damage, so our follow-up period is concise (no more than three years).

Future studies with larger cohorts and more extended follow-up periods will be necessary to validate the results obtained, and allow an association between the BAFF system molecules and graft survival. Potential variables could influence the transcripts, and have not been evaluated, such as the type of induction therapy, dose of immunosuppression, or the influence of infections on KTRs.

In conclusion, monitoring the expression of BAFF receptors in a transplanted patient could help the pathologist to predict future allospecific humoral responses in kidney transplantation, with the ability to predict graft rejections to such an extent that the necessary clinical measures can be taken to save grafts and increase the quality and life expectancy of the transplanted patient.

## Figures and Tables

**Table 1:** Demographic data and clinical characteristics of kidney recipient patients’ demographic data and clinical characteristics included in the BAFF system gene expression study.

	NAR (*n* = 35)	AR (*n* = 5)	*p* ^a^
Age (years)	56.1 ± 1.59	60.0 ± 6.71	0.358
Gender (male/female) *n*/(%)	19 (54.3)/16 (45.7)	3 (60)/2 (40)	1.000
HLA mismatches ^b^	4.1 ± 0.17	4.5 ± 0.64	0.524
Live donor (%)	2 (5.7)	1 (20)	0.338
Preformed anti-HLA antibodies (%)	6 (17.1)	1 (20)	1.000
Induction therapy (Tim/Bas)	11 (31.4)/5 (14.3)	3 (60)/0 (0)	0.386
Delayed graft function (%)	8 (23.5)	2 (40)	0.279
Type of rejection (cellular/humoral)	-	3 (60)/2 (40)	-

Tim, thymoglobulin; Bas, basiliximab; NAR, non-acute rejection; AR, acute rejection. SD, standard deviation. Quantitative data were expressed as the mean value ± SD. ^a^ Comparisons were made using Fisher’s exact test or X^2^ for qualitative variables, and the non-parametric Mann–Whitney U test for quantitative variables. Values of *p* < 0.05 were considered significant. ^b^ Total differences between donor and recipient for the HLA-A, HLA-B, and HLA-DRB1 genes.

## Supplementary Materials

The following supporting information can be downloaded at: https://www.mdpi.com/article/10.3390/jcm11143956/s1, Table S1: Characteristics of the gene expression studies obtained from the GEO database used in the meta-analysis.

## Abbreviations

B-cell activating factor (BAFF); a proliferation-inducing ligand (APRIL); BAFF receptor (R-BAFF); transmembrane activator and calcium modulator, and cyclophilin ligand interactor (TACI); B-cell maturation antigen (BCMA).
