# Supplementary material for: Evaluating the Link between BAFF System Gene Expression and Acute Rejection Development in Kidney Transplantation
Source: J Clin Med. 2022 Jul 7;11(14):3956. doi: 10.3390/jcm11143956 (PMC9319040; doi:10.3390/jcm11143956)
Supplement: Supplementary file 1 [file jcm-11-03956-s001.zip › jcm-1795001-supplementary.pdf]

Supplementary Table S1. Characteristics of the gene expression studies obtained from the GEO database used in the meta-analysis.

**ID Study**	**Platform**	**Type of sample**	**Number of samples (NAR/AR)**	**Ref.**
GSE14346	Affymetrix Human Genome U133 Plus 2.0 Array	SP	40/30	[26]
GSE15296	Affymetrix Human Genome U133 Plus 2.0 Array	SP	46/51	[27]
GSE46474	Affymetrix Human Genome U133 Plus 2.0 Array	SP	20/20	[28]
GSE36059	Affymetrix Human Genome U133 Plus 2.0 Array	B	281/122	[29]
GSE21374	Affymetrix Human Genome U133 Plus 2.0 Array	B	206/76	[30]

SP, Peripheral Blood; B, Biopsy; NAR, Non-Acute Rejection; AR, Acute Rejection
